# News and Events of the Week

**Published:** 1911-07-22

**Authors:** 


### NEWS AND EVENTS OF THE WEEK.

"The Literary Pageant" is a collection of short stories, sketches, and illustrations by well-known writers and artists, edited by Mr. Stanley Portal-Hyatt, and published in aid of the Teck Fund for the Middlesex Hospital by T. Werner Laurie, of Fleet Street. The price is only one shilling, and the contents of the compilation are so good that the expenditure must be considered as an excellent investment by those who delight in such literary efforts.

"Medical Homes for Private Patients," the sixpenny directory of medical and nursing homes which is annually issued by The Scientific Press, 28 and 29 Southampton Street, Strand, is an informative and very complete little reference handbook. It gives full details on all points that intending patients may desire to know about. The classified list of medical and surgical consultants is very complete, and should prove of value to those who wish to select an adviser from the host of specialists practising in London.

The Société de l'internat des Hôpitaux de Paris has decided to open a subscription with a view to the erection of a monument to the memory of Professor Guinard, a victim of duty, recently assassinated by one of his former patients at the Hôtel Dieu. The Society has also stated that if sufficient funds are forthcoming a "prix Guinard" will be founded. Subscriptions should be sent to Dr. Jayle, general secretary of the Society, 238 Boulevard St. Germain, Paris. The monument will be erected in the Court of Honour of the Hôtel Dieu.

We have received from Messrs. John Wright and Sons, the well-known Bristol medical publishers, copies of their new "First-Aid Charts" which have been compiled by Dr. Edward Ibotson. These charts are thoroughly reliable and accurate so far as the essentials of first-aid are concerned, and should prove of immense assistance to lecturers and classes. There are four sets, and the whole deals with every imaginable point in ordinary first-aid work that the student can desire information about. The charts are well printed, attractive, and sold at the low price of 6d. per sheet, or 1s. 6d. for the whole set of four.

The eleventh voyage for medical studies, Voyage d'Etudes Médicales, will take place from August 28 to September 11, 1911, under the presidency of Professor Landouzy. It will comprise the watering-places of the South-east of France visited in the following order: Vals, Montmiral, Balaruc-les-Bains, Lamalou, Alet, La Fou-Saint-Paul-de-Fenouillet, Prats-de-Mollo, La Presle, Amélié-les-Bains, Le Boulou, Banyuls-sur-Mer, Molitg, Le Vernet, Thuès, Mont-Louis, Font-Romeu, Les Escaldes, Ax-les-Thermes, Ussat, Aulus, Salies-du-Salat. A reduction of half price will be allowed on all the railways from the place of residence of the member to the starting-point, Lyons. Foreign doctors will benefit by this reduction from the station at which they land on French soil. The same reduction is made for the journey home from the last stopping-place, Toulouse. The charge for the whole itinerary from Lyons to Toulouse is 350 francs (£14), which includes first-class fare on the railway, hotel accommodation, board, luggage dues, and tips. For information and the payment of subscriptions apply to Dr. Carron de la Carrière, 2 rue Lincoln, Paris, or to Dr. Jouaust, 4 rue Fréderic-Bastiat, Paris.

"Sunny Rhyl" is the title of the illustrated guide-book to this bracing seaside resort. It gives a large amount of information regarding the attractions of the place, which fully deserves the attention of those in search of a holiday resort which is up to date, sanitary, and from a health point almost ideal.

On the initiative of the French Public Health Authority, a committee, under the presidency of Professor Gariel, and consisting of oculists, physicists, and engineers, has been appointed to study natural and artificial lighting from the point of view of ocular and general hygiene. The committee is also to examine into the various photometric methods and to fix the amount of light compatible with normal working of the eye.

One of the most readable of railway books—the sort of literature one takes into the carriage—is "Queer Stories," republished from *Truth* for the seventeenth time in succession. It is an amazing shillingsworth, for every story, however absurd and far-fetched it may be (as, for instance, that very absurd narrative of a doctor man who "spoofed" his friend into imagining a pretended valvular affection of the heart that was inevitably deadly in the course of a few years!), is excellently written, and interesting because it has a definite situation and plot. The book is to be heartily commended to everyone who wants something light and amusing wherewith to while away a lazy hour.

The following are being invited to accept the honorary degree of LL.D. of Birmingham University: Mr. John Burns, M.P., Sir Francis Lovell (President of the Tropical Medicine Section), Dr. R. H. Chittenden (Professor of Physiology at Yale), Professor H. Oppenheim (Neurologist of Berlin), Professor Paul Strassman (Assistant Professor of Obstetrics, Berlin), Dr. Byron Bramwell (President Royal College of Physicians, Edinburgh), Dr. J. A. Macdonald (Chairman of Council, British Medical Association), Dr. R. A. Reeve (Professor of Ophthalmology at Toronto), and Professor Sims Woodhead (Professor of Pathology at Cambridge).

